# Connecting high‐throughput biodiversity inventories: Opportunities for a site‐based genomic framework for global integration and synthesis

**DOI:** 10.1111/mec.15797

**Published:** 2021-02-02

**Authors:** Paula Arribas, Carmelo Andújar, Martin I. Bidartondo, Kristine Bohmann, Éric Coissac, Simon Creer, Jeremy R. deWaard, Vasco Elbrecht, Gentile F. Ficetola, Marta Goberna, Susan Kennedy, Henrik Krehenwinkel, Florian Leese, Vojtech Novotny, Fredrik Ronquist, Douglas W. Yu, Lucie Zinger, Thomas J. Creedy, Emmanouil Meramveliotakis, Víctor Noguerales, Isaac Overcast, Hélène Morlon, Alfried P. Vogler, Anna Papadopoulou, Brent C. Emerson

**Affiliations:** ^1^ Island Ecology and Evolution Research Group Instituto de Productos Naturales y Agrobiología (IPNA‐CSIC) San Cristóbal de la Laguna Spain; ^2^ Department of Life Sciences Imperial College London London UK; ^3^ Comparative Plant and Fungal Biology Royal Botanic Gardens London UK; ^4^ Section for Evolutionary Genomics, Faculty of Health and Medical Sciences, Globe Institute University of Copenhagen Copenhagen Denmark; ^5^ Université Grenoble Alpes, CNRS, Université Savoie Mont Blanc LECA, Laboratoire d’Ecologie Alpine Grenoble France; ^6^ School of Natural Sciences Bangor University Gwynedd UK; ^7^ Centre for Biodiversity Genomics University of Guelph Guelph Canada; ^8^ School of Environmental Sciences University of Guelph Guelph Canada; ^9^ Centre for Biodiversity Monitoring (ZBM) Zoological Research Museum Alexander Koenig Bonn Germany; ^10^ Department of Environmental Sciences and Policy University of Milano Milano Italy; ^11^ Department of Environment and Agronomy INIA Madrid Spain; ^12^ Biodiversity and Biocomplexity Unit Okinawa Institute of Science and Technology Graduate University Onna‐son Japan; ^13^ Department of Biogeography Trier University Trier Germany; ^14^ Aquatic Ecosystem Research, Faculty of Biology University of Duisburg‐Essen Essen Germany; ^15^ Centre for Water and Environmental Research (ZWU) Essen University of Duisburg‐Essen Essen Germany; ^16^ Biology Centre, Institute of Entomology Czech Academy of Sciences Ceske Budejovice Czech Republic; ^17^ Faculty of Science University of South Bohemia Ceske Budejovice Czech Republic; ^18^ Department of Bioinformatics and Genetics Swedish Museum of Natural History Stockholm Sweden; ^19^ State Key Laboratory of Genetic Resources and Evolution Kunming Institute of Zoology, Chinese Academy of Sciences Kunming China; ^20^ Center for Excellence in Animal Evolution and Genetics Chinese Academy of Sciences Kunming China; ^21^ School of Biological Sciences University of East Anglia Norwich UK; ^22^ Institut de Biologie de l’ENS (IBENS), Département de biologie, École normale supérieure, CNRS, INSERM Université PSL Paris France; ^23^ Department of Life Sciences Natural History Museum London UK; ^24^ Department of Biological Sciences University of Cyprus Nicosia Cyprus; ^25^ Division of Vertebrate Zoology American Museum of Natural History New York USA

**Keywords:** biodiversity assessment, DNA metabarcoding, Genomic Observatories, harmonized data generation, high‐throughput sequencing

## Abstract

High‐throughput sequencing (HTS) is increasingly being used for the characterization and monitoring of biodiversity. If applied in a structured way, across broad geographical scales, it offers the potential for a much deeper understanding of global biodiversity through the integration of massive quantities of molecular inventory data generated independently at local, regional and global scales. The universality, reliability and efficiency of HTS data can potentially facilitate the seamless linking of data among species assemblages from different sites, at different hierarchical levels of diversity, for any taxonomic group and regardless of prior taxonomic knowledge. However, collective international efforts are required to optimally exploit the potential of site‐based HTS data for global integration and synthesis, efforts that at present are limited to the microbial domain. To contribute to the development of an analogous strategy for the nonmicrobial terrestrial domain, an international symposium entitled “Next Generation Biodiversity Monitoring” was held in November 2019 in Nicosia (Cyprus). The symposium brought together evolutionary geneticists, ecologists and biodiversity scientists involved in diverse regional and global initiatives using HTS as a core tool for biodiversity assessment. In this review, we summarize the consensus that emerged from the 3‐day symposium. We converged on the opinion that an effective terrestrial Genomic Observatories network for global biodiversity integration and synthesis should be spatially led and strategically united under the umbrella of the metabarcoding approach. Subsequently, we outline an HTS‐based strategy to collectively build an integrative framework for site‐based biodiversity data generation.

## 1 INTRODUCTION

High‐throughput sequencing (HTS) is increasingly being used for the characterization and monitoring of ecosystems and holds the prospect for a much deeper understanding of global diversity on Earth (e.g. Bohan et al., 2017; Bush et al., 2017; Taberlet et al., 2018). Deep DNA sequencing of biodiversity conducted at particular sites (i.e., site‐based biodiversity assessment) presents opportunities for both detailed characterization and monitoring of local biomes. If replicated across other sites, broader scale biodiversity patterns/trends may emerge. Studying these trends not only allows better understanding of the processes that generate them, but is also highly relevant for (i) developing conservation and management policies, (ii) mapping living resources to allocate funding and efforts to biodiversity research, and (iii) comparing and understanding ecosystems (Pedrós‐Alió & Manrubia, 2016). We may be witnessing the emergence of a new scientific paradigm, in which the conjunction of massive publicly available data sets and computational power lead to patterns and underlying causes being sought directly, rather than through more traditional hypothetico‐deductive methods (Hey et al., 2009). If such a transition is to occur in biodiversity science, HTS can play a central role. The universality, reliability and efficiency of HTS data can potentially facilitate linking data among assemblages from almost anywhere (including elusive domains referred to as last biotic frontiers), at multiple biological levels (genes, populations, species, lineages), for any taxonomic group and regardless of prior taxonomic knowledge (Bik et al., 2012; Deiner et al., 2017; Ji et al., 2013). Projects from local to global scales are currently generating massive quantities of biodiversity inventories (i.e., community‐level data) derived from HTS. These offer unprecedented opportunities to better understand both the distribution and the dynamics of biodiversity, and to propose strategies for its conservation. However, collective international efforts are required to optimally facilitate global integration and synthesis. While integrative frameworks for site‐based genomic science do exist in the microbial realm (e.g., Gilbert et al., 2010, 2014), such frameworks have yet to be extended to nonmicrobial biotas.

From the November 11 to 13, 2019, evolutionary geneticists, ecologists and biodiversity conservationists involved in diverse large‐scale initiatives based on HTS biodiversity assessments met at the University of Cyprus for the Next Generation Biodiversity Monitoring Symposium. Funded through iBioGen, a European Commission‐funded (H2020) project, the symposium was conceived to discuss how best to develop an integrative HTS framework for measuring and understanding global patterns of biodiversity. The main objectives were to: (i) assess the current state of site‐based biodiversity science using DNA‐sequence information; and (ii) identify the challenges and opportunities for implementing a network for global integration and synthesis, featuring HTS as a core tool and with particular emphasis on the nonmicrobial terrestrial realm. In this review we synthesize the diversity of ideas raised over the 3‐day symposium, and discuss their relevance for a more integrative genomics‐informed biodiversity science.

## 2 DNA SEQUENCE DATA: FROM GLOBAL REPOSITORIES TO GENOMIC OBSERVATORIES FOR INTEGRATION AND SYNTHESIS IN BIODIVERSITY SCIENCE

The systematic archiving of DNA‐sequence data within global molecular repositories by the International Nucleotide Sequence Database Collaboration (DDBJ, EMBL‐EBI and NCBI) emerged from the need to guarantee the preservation of, and accessibility to, genetic sequence data, a universal data type that links all biodiversity. These public repositories have the potential to serve as reference databases, providing the bedrock for global integration and synthesis in biodiversity science, in a similar fashion and complementary to other repositories focused on species occurrence records (e.g., GBIF, http://www.gbif.org/). Although the potential of DNA sequence repositories to document global patterns of genetic and phylogenetic diversity has been demonstrated (Holt et al., 2013; Miraldo et al., 2016), important limitations have been identified, primarily linked to: (i) a lack of standardized metadata associated with DNA sequences and (ii) taxonomic, geographical and sampling biases associated with these public repositories (Deck et al., 2017; Pope et al., 2015; Yilmaz et al., 2011).

The Barcode of Life Data Systems (BOLD; Ratnasingham & Hebert, 2007; http://boldsystems.org/) emerged as a curated workbench for the storage of specific DNA sequences under the umbrella term DNA barcoding (Hebert et al., 2003), addressing, in part, the metadata gap within global molecular repositories. The value of a taxonomically accurate and georeferenced DNA sequence database, even if focused on a single locus, has been demonstrated by analyses revealing how genetic diversity is structured at multiple scales (deWaard et al., 2019; Muñoz, 2007; Theodoridis et al., 2020). Additionally, numerous local–regional studies have benefitted from the contextualization of local data across larger spatial scales (Ashfaq et al., 2017; Cicconardi et al., 2017). More recently, the Genomic Observatories Metadatabase (GEOME; http://www.geome‐db.org/) has been provided as an open access repository for metadata associated with genetic data (or biosamples) of any type, linking ecologically and evolutionarily relevant metadata with publicly archived DNA sequence data (Deck et al., 2017; Riginos et al., 2020). GEOME thus provides a data management workflow to facilitate the use of public molecular repositories for global integration and synthesis.

Developments to address the metadata gap linked to genetic data are encouraging, but most records within public repositories are incidental point records (i.e., single specimens) that lack information about co‐observed species (Jetz et al., 2019). This also imposes important constraints for documenting global biodiversity patterns, even if such point estimates are well contextualized with metadata. These constraints are comparable to those described for the GBIF database (Faith et al., 2013), including important biases for taxonomic and sampling coverage that are difficult to resolve in the short to medium term with additional point records. Inventories (i.e., site‐based recording of multiple species) are a key biodiversity data type harbouring much potential for model‐supported assessment of spatial biodiversity and its change (Jetz et al., 2019). However, the use of site‐based data for the assessment of biodiversity distribution and change has been more a promise than a reality until recently (but see Phillips et al., 2019; van den Hoogen et al., 2019), and further efforts on data generation, standardization, integration and re‐use are needed (Guralnick et al., 2018; König et al., 2019). In the case of DNA sequence data, HTS provide the potential to shift from incidental observations toward biodiversity inventories by (i) generating community‐scale biodiversity data in a consistent and comparable manner; (ii) stimulating development of automated processing pipelines; and (iii) radically expanding taxonomic coverage, and thus increasing potential for synthetic analyses of global biodiversity (Bush et al., 2017, 2019). A pioneering international effort to generate site‐based molecular information for global biodiversity synthesis has been focused on microbes, primarily by the Earth Microbiome Project (EMP; Gilbert et al., 2010, 2014; https://earthmicrobiome.org). The EMP was founded in 2010, with the core objective to construct a global microbial map, and has been catalysed by the explosion of metagenomics studies characterizing microbiomes worldwide. It has grown exponentially, now comprising a collaborative network of more than 500 researchers from 161 international institutions. It provides protocols and standards, and supports prepublication data sharing and crowd‐sourcing data analysis to enable universal patterns of microbial diversity to be explored (Shoemaker et al., 2017; Thompson et al., 2017).

In an analogous initiative to the EMP, but one which extends to complete ecosystems, Davies et al. (2012) advocated the establishment of an international network of Genomic Observatories (GOs), with the subsequent publication of a founding charter (Davies et al., 2014). Site‐based genomic inventories were proposed as a fundamental data source for biodiversity science, but with less focus on broad spatial coverage. Rather, it was suggested that ecological understanding could advance by focusing massive sequencing efforts on a limited number of model ecosystems. Concept definitions within the founding charter of the GO network are as follows. A GO is defined as an ecosystem or site subject to long‐term scientific research, including (but not limited to) the sustained study of genomic biodiversity (the genetic variation found among genomes) from single‐celled microbes to multicellular organisms. A GO network is a network of sites that fall within the GO definition, although the fuller definition incorporates researcher and institutional details (see Davies & Field, 2012; Davies et al., 2012, 2014). These definitions provide an abstract concept of site‐based biodiversity measurement with three sampling dimensions: (i) genomic, (ii) temporal and (iii) spatial. The GO network ambition of a genome‐centred characterization of biodiversity across both space and time is exciting. However, the GO concept itself is greatly ambitious, which may in part explain its limited adoption being restricted, so far, to initiatives primarily focused on marine meiofauna (Buttigieg et al., 2019; Kopf, 2015). The focus of the iBioGen meeting was to review the GO network concept and vision within the current state of the art and ongoing initiatives, and identify tangible opportunities to catalyse a site‐based genomic network for global integration and synthesis of biodiversity data in the terrestrial realm.

## 3 TOWARD A SITE‐BASED TERRESTRIAL GENOMIC NETWORK FOR INTEGRATIVE AND SYNTHETIC BIODIVERSITY SCIENCE

Three major challenges have been identified for the implementation of GOs (Davies et al., 2012): (i) logistical constraints associated with field sampling, (ii) sequencing and data curation costs, and (iii) the collection of relevant metadata. In relation to metadata, it was sensibly suggested that GOs should integrate within existing research sites that are already rich in metadata. However, the logistical and financial challenges associated with field sampling and genomic data generation remain as much a limitation now as they were 8 years ago. Even if sequencing costs were to dramatically decrease, and technology were to dramatically improve, the ambition of the original GO concept would still remain beyond reach in the short to medium term. However, we believe that implementation of the conceptual framework of Davies et al. (2012, 2014) can be achieved with a realistic appreciation of logistical and financial constraints, and an attempt to integrate the three principal axes of the concept: space, time and genome.

### 3.1 The spatial and temporal dimensions

Achieving a site‐based genomic network, capable of providing data for global syntheses in biodiversity science, will require trade‐offs with regard to the axes of space, time and genome. This is because integration across the three axes will vary depending on the question being asked. For example, estimating and mapping the number of species on Earth (*the Linnean challenge*), including taxonomic, phylogenetic and functional diversity, places heavy emphasis on the spatial axis. Taxonomic information can be achieved with limited investment along the genomic axis (e.g., via metabarcoding or future alternatives), with more investment for phylogenetic information, and a much greater effort to examine functional diversity. Addressing the global estimation of species distributions and ecological niches (*the Wallacean challenge*) also falls most heavily along the spatial axis. Spatial sampling is important not only to tease apart the population‐ and community‐level processes that impact macroecological and macroevolutionary patterns, but also to develop predictive models for the impact of global change on biodiversity and ecosystem functioning. The temporal axis is also particularly important here, as it can directly inform about resistance, resilience and dynamics of community change in the face of natural and anthropic disturbance. However, time series are extremely difficult to obtain and under some conditions time can even be substituted for space, such as using sediment cores across glacial retreat areas (Chen & Ficetola, 2020) or geologically dated chronosequences (Rominger et al., 2016).

The spatial dimension of GOs was implicit within the original definition of Davies et al. (2012) and was explicitly incorporated within the concept of the GO network (Davies et al., 2014). However, the genomic and temporal dimensions were given greater emphasis, with the spatial dimension assuming secondary importance. GOs were framed in the context of long time series for particular sites, or so‐called “model ecosystems.” Despite this earlier temporal emphasis, our discussions concluded that a sustained and coordinated effort is most likely to emerge through opportunities for a spatially based network, which will provide more immediate and broader opportunities for global integration. Site‐based research across the ecoregions could efficiently integrate data into the Global Earth Observation System of Systems (http://www.geoportal.org/), one of the main objectives of the GO network as initially defined (Davies et al., 2014). Recent developments open the door to connect Earth Observation (EO) technologies to biodiversity and ecosystems (CEOBE; Bush et al., 2017) to derive broader scale correlations and inferences from continuous remote‐sensing data with point‐sample biodiversity data. This approach is anchored in the generation of site‐based biodiversity data, which are then combined with EO variables to interpolate continuous maps of biodiversity variables. The generation of spatially dense, site‐based biodiversity data across ecologically distinct regions is fundamental for parameterization within the model of Bush et al. (2017), and they propose HTS as a universally applicable tool for this purpose. We believe that a collaborative site‐based genomic network, where spatial coverage is the primary focus, is a realistic ambition that bridges both the GO network and CEOBE visions to model continuous surfaces of biodiversity across whole regions, and eventually the planet.

We also see a more fundamental argument for a network of shallow (i.e., without extensive effort on the temporal axis) sampling points. Limited logistical constraints associated with shallow sampling mean that implementation is less prohibitive, enhancing the opportunity for independent uptake, and the development of a globally extensive network. We converged in opinion that the most effective way to develop a GO network is to build upon and integrate among existing initiatives using HTS to generate inventory data at multiple spatial scales (see below for details). Additionally, international efforts for global microbial biodiversity syntheses, such as the EMP (Gilbert et al., 2014), the Ocean Sampling Day consortium (OSD; Kopf, 2015) and TARA Oceans (Karsenti et al., 2011), align with a spatially led vision for a GO network. These may serve as models for the development of best practices, methodological harmonization and analytical synthesis.

Reduced logistical challenges associated with data generation for a spatially led vision of the network are also likely to have positive statistical consequences. We see higher spatial replication as potentially reducing the degree of data harmonization required for global integration and comparison. Coverage for a large number of shallow sampled sites can correct for local‐scale noise that would be more difficult to account for with a network of fewer temporally sampled sites. Finally, a GO network where shallow sampling of biodiversity is a cornerstone is a potential catalyst for a noncentralized, bottom‐up network expansion, with potential for citizen‐science to collect site‐based biodiversity samples over large areas (e.g., Kopf, 2015).

### 3.2 The genomic dimension

The concept of a GO network was originally envisaged as community‐scale genome‐level data capture, from single‐celled microbes to multicellular organisms, through time (Davies et al., 2012, 2014). We agreed that this ambition of characterizing ecosystems at the deepest genomic dimension (whole‐genome scale) is currently unrealizable, particularly within a spatially dense sampling framework for biodiversity synthesis. In the light of this, we sought to define a more plausible integration of genome sequencing technology and genome data within a global network for biodiversity observation. Agreement was reached that less exhaustive genomic sampling approaches can provide foundational site‐based biodiversity information, where data standards can be relatively easily implemented. Of high importance is that relative cost efficiency helps to democratize implementation, facilitating network expansion in economically limited tropical countries, which are megadiverse, often under greater threats and much less sampled (Zinger et al., 2020).

A single‐locus approach represents the lowest level of genomic investment for comparative site‐based biodiversity assessment. DNA barcoding is a simple idea wherein short genomic regions that typically differ between species can be used to assign some level of taxonomic identity to an unknown specimen (Hebert et al., 2003). The extension of this method to more complex samples where multiple specimens/species are present has given rise to the term DNA metabarcoding (Taberlet et al., 2012; Yu et al., 2012) involving massively parallelized HTS characterization of taxonomic composition within a biological sample. This reduces the logistical and the financial costs associated with individualized sampling and sequencing of specimens (Ji et al., 2013). These are transversal benefits that are particularly relevant for hyperdiverse assemblages such as those from soil (Arribas et al., 2016) or tropical canopies (Creedy et al., 2019). Individualized processing of specimens from such assemblages is a major bottleneck, and as such they are recurrently ignored in conventional biodiversity surveys. Metabarcoding can additionally be deployed to inventory diversity from degraded tissues or without direct sampling of organisms, as exemplified by “environmental DNA” (eDNA; Deiner et al., 2017) and “invertebrate‐derived DNA” (Schnell et al., 2012), thus maximizing diversity recovery from biosamples.

DNA metabarcoding involves PCR (polymerase chain reaction)‐coupled HTS of one or more DNA barcode markers, directly from bulk or environmental samples, represents the most cost‐efficient approach for obtaining molecular community profiles (Porter & Hajibabaei, 2018), and is already established as a reference tool to map global microbial biodiversity (e.g., Karsenti et al., 2011; Kopf, 2015). It is highly scalable within a relatively simple methodological framework, facilitating increased sampling breadth and depth within biodiversity studies. Importantly, it offers many opportunities for standardization, reproducibility and sample handling efficiency.

Metabarcoding can generate biodiversity inventory data in which taxonomy can be assigned at a sufficiently informative taxonomic rank for the reliable, relative estimation of richness and composition of communities. Conventional processing of raw metabarcoding data clusters sequences based on their similarity, resulting in operational taxonomic units (OTUs), for which taxonomic assignment to species is possible when reference barcode sequences are available. However, even without species‐specific reference libraries, assignment to some taxonomic level can be achieved using public repositories (e.g., GenBank, BOLD, SILVA and UNITE). The fundamental output of metabarcoding is ecological tables of samples by taxa (OTUs), with the key advantage that this can be achieved at an unprecedented scale and cost‐efficiency. By generating species inventories across a network of sites, metabarcoding data can be used to estimate ecological/trophic networks through co‐occurrence analysis (Bohan et al., 2017). Although there are important limitations associated with inferring interactions from co‐occurrence data (Blanchet et al., 2020), geographically extensive data can provide hypothetical frameworks within which more directed sampling and sequencing (e.g., by the analyses of gut contents, Alberdi et al., 2019), can be undertaken within particular GOs (see Box 1).

Box 1 ***SuperGOs*: extending the temporal and genomic axes of the GO network**

**FIGURE 1:**
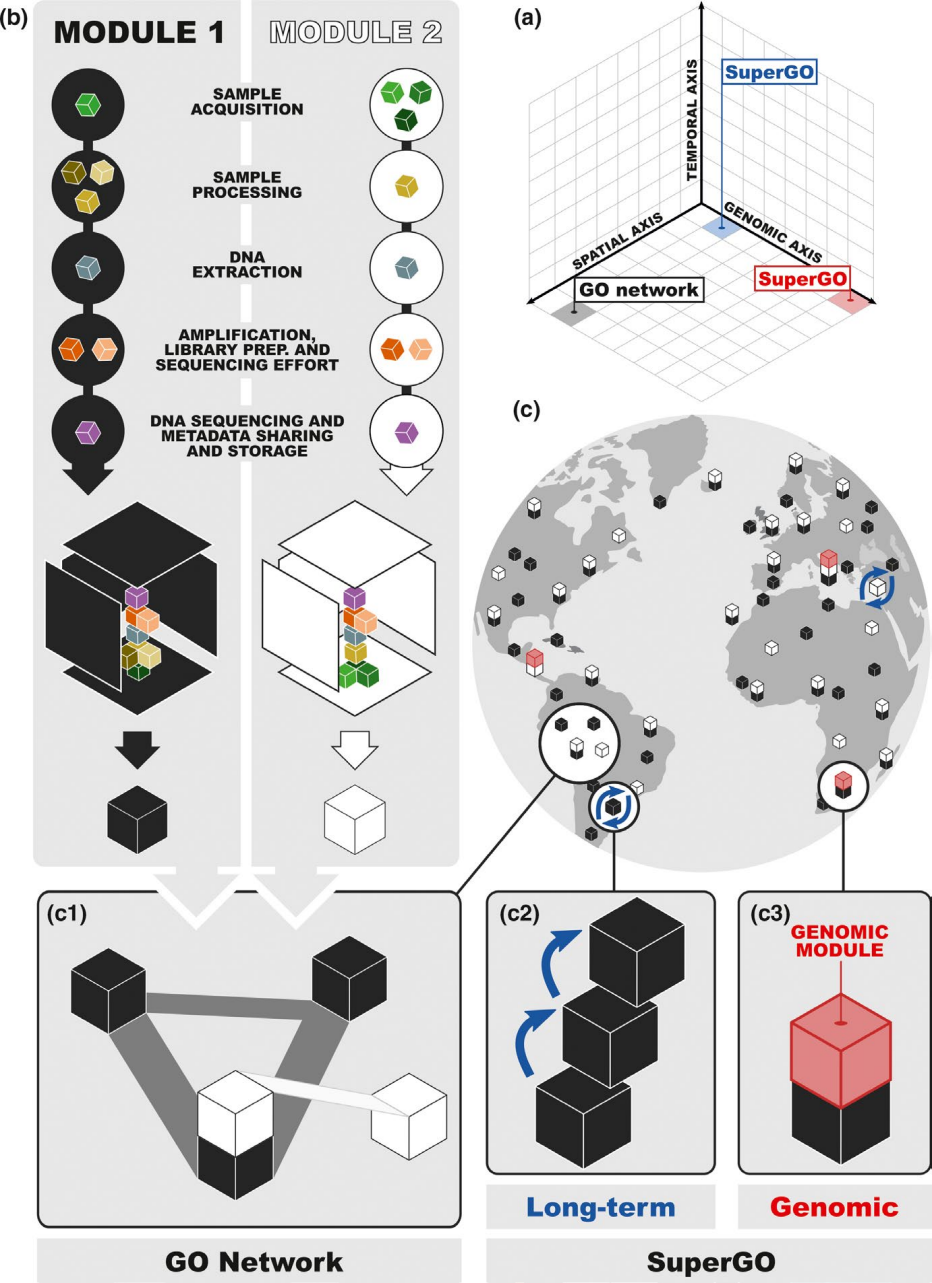
Conceptual representation of the modular framework for harmonized data generation within a spatially led Genomic Observatories (GOs) network. (a) Representation of the three principal axes of the GO concept: space, time and genome. (b) Representation of the modular structure for protocol harmonization across GOs. Modules, as basic building blocks of the framework, can provide simple, integrated and interoperable procedures for site‐based characterization of extensive biodiversity fractions. Black block: module 1 (e.g., soil biodiversity); white block: module 2 (e.g., terrestrial arthropods); coloured blocks: protocols or submodules across the sequence of the five steps for a harmonized generation of inventory data within each module. (c) Map showing how modules can be implemented in: (c1) a spatially led network of sites for biodiversity inventory with shallow temporal and genomic efforts (spatial axis for the GO network concept); (c2) replicated through time within a site by repeated module implementation or historical samples (temporal axis for the SuperGO concept); and (c3) complemented with genomic resources (e.g., partial and complete genomes, microbiomes, gut contents) for specimens/assemblages within a site (genomic axis for the SuperGO concept)

Within the spatially led terrestrial GO network that we propose, where metabarcoding is at the core of data generation, both the temporal and the genomic axes can be more deeply sampled (Figure 1), consistent with the idea of “model ecosystems” (Davies et al., 2012, 2014). As well as potentially providing a deeper understanding of dynamics at a local scale, these additional layers of information may also serve as: (i) calibrations within the global network (e.g., for the assessment of inventory completeness, validation of diversity estimations using different sources of genomic information); (ii) sites where the interplay between different dimensions of diversity (e.g., genetic, taxonomic, phylogenetic, functional, interaction) can be assessed in depth; or (iii) sites where the periodic implementation of a particular module or modules could be undertaken to generate long‐term HTS biodiversity data series. Due to the more intense nature of data generation associated with such sites, we refer to them as “SuperGOs.” Temporal sampling could take advantage of sites with rich histories of data collection (e.g., sites within the Long‐Term Ecosystem Research network, https://www.ilter.network/; or ICP Forests, http://icp‐forests.net), or existing national biomonitoring frameworks (e.g., the UK Countryside Survey, https://countrysidesurvey.org.uk/; the German Environmental Specimen Bank, https://www.umweltprobenbank.de/en). Alternatively, sites with natural temporal records, such as lake sediment cores (e.g., Chen & Ficetola, 2020; Pansu et al., 2015) or organic deposits (e.g., in feco‐urinary middens, Murray et al., 2012), may provide historical context for more extensive forward temporal sampling (reviewed in Bálint et al., 2018). SuperGOs could also be a focus for generating genomic resources (i.e., partial or complete genomes, microbiomes, diet) for well‐characterized taxa, or species of specific interest within sites. For example, a series of spatially clustered sites, well characterized for their terrestrial arthropod fractions of biodiversity with metabarcoding, could be used to provide broader context for understanding how habitat structure, prey availability and feeding preferences influence vertebrate insectivores (Makiola et al., 2020). Increasing the depth of genomic sampling within a site, from barcode regions, to whole organelle genomes, to partial nuclear genomes and finally to whole nuclear genomes, will in itself generate new resources that may promote further synergy. As an example, for any annotated genome produced within a SuperGO, the associated barcode sequence metadata for that species allow its presence to be easily established in any other GO. If pertinent (e.g., due to inter‐GO variation for climatic or biotic variables), targeted gene sequencing or whole genome resequencing could be implemented in additional GOs to explore potential adaptive variation.

While we reached a consensus that metabarcoding is the current candidate tool for a GO network, limitations were also recognized. Deriving abundance information from metabarcoding data remains a challenge, primarily due to inherent biases in the PCR step, but also because of variation in gene copy number, organelle number, and technical aspects of sampling, laboratory, sequencing and bioinformatic workflows (Deagle et al., 2019; Taberlet et al., 2018; Zinger et al., 2019). Generally speaking, it has been suggested that the most conservative approach is to treat DNA metabarcoding data as presence–absence data after applying a high filtering threshold based on read relative abundance to mitigate the impact of the false positives (Elbrecht et al., 2015; Taberlet et al., 2012). For general patterns of community assembly, it has been shown that ecological indices using abundance or presence‐only data perform similarly well (Beentjes et al., 2018; Creedy et al., 2019; Ranasinghe et al., 2012). Considering read abundance as a probability of presence has also been shown to provide alpha/beta diversity estimates that are closer to those obtained with traditional approaches (Calderón‐Sanou et al., 2019). The use of taxon‐specific correction factors and occupancy models shows great promise for improving abundance estimates from metabarcoding data (e.g., Kembel et al., 2012; Krehenwinkel et al., 2017) and for some taxa it has been shown that read abundance can be used to estimate actual abundance (Schenk et al., 2019). However, this requires high taxon‐specific effort, and thus implementation will probably be limited with regard to complex and/or hyperdiverse communities, at least in the near future.

As well as recording species presence, metabarcoding can potentially provide a measure of haplotype variation within and across communities. Recent advances in the removal of both PCR and sequencing errors (Amir et al., 2017; Callahan et al., 2016; Edgar, 2016), and co‐amplified nuclear copies of mitochondrial genes (Andújar et al., 2020), open the door for bypassing sequence clustering steps, thus yielding haplotype‐level data (amplicon sequence variants) for downstream analyses (Elbrecht et al., 2018; Turon et al., 2020). Thus, alpha and beta diversity can be analysed at different hierarchical levels to understand how population‐ and community‐level processes drive macroecological and macroevolutionary patterns (Arribas et al., 2020; Tsuji et al., 2020). This possibility of recording data in the form of haplotype‐level community tables would improve comparability of biodiversity surveys from independent studies. This is because individual haplotypes are directly comparable between independently processed data sets, while OTUs are not (Callahan et al., 2017). We thus believe that appropriately denoised haplotype tables would be a substantial step in integration among independent metabarcoding data sets.

Metabarcode data provide, to some extent, phylogenetic information among community members, and this has been exploited to reveal phylodiversity patterns (alpha and beta) across communities, mostly in prokaryotes (e.g., Fierer & Jackson, 2006; Goberna et al., 2016), and evolutionary processes across entire lineages (e.g., diatoms, Lewitus et al., 2018). We recognize the inherent limitations of short metabarcode sequences for phylogenetic resolution, but note that this can be partly overcome by placing metabarcode sequences in backbone phylogenies (Balaban et al., 2018; Pérez‐Valera et al., 2018). A phylogenetic framework can simultaneously (i) assist species delimitation and taxonomic assignment, through the phylogenetic placement of the massive quantities of unidentified OTUs/haplotypes, and (ii) contribute tip‐level data to the Tree of Life (Hinchliff et al., 2015). We therefore see potential for synergy between a GO network built upon community‐level metabarcoding, and other global initiatives developing phylogenomic (e.g., the Earth Biogenome Project, Lewin et al., 2018) and taxonomic resources (e.g., BOLD, Ratnasingham & Hebert, 2007). Organelle skimming of mixed community samples can directly provide the phylogenetic context of a local sample (Crampton‐Platt et al., 2015) which, when combined with samples from other sites, each contribute to an increasingly complete Tree of Life (e.g., SITE‐100 project; www.site100.org). Such efforts to develop taxonomically curated and phylogenetically defined reference specimen repositories could be undertaken within SuperGOs (see Box 1).

With currently available DNA sequencing technology, other molecular techniques for site‐based biodiversity analysis remain uncompetitive compared to metabarcoding, when simultaneously considering scalability, cost and tractability (Porter & Hajibabaei, 2018). Metagenomics (Handelsman et al., 1998) and metatranscriptomics (Poretsky et al., 2005) present a key advantage over metabarcoding for characterizing community samples, as their PCR‐free protocols remove biases associated with the PCR amplification step of metabarcoding, thus making abundance estimates less challenging (e.g., Tang et al., 2015). These techniques sequence all DNA/RNA in a sample, and sequences are then interrogated *in silico* for taxonomically and functionally informative gene sequences. However, for fundamental tasks of estimating species richness and taxonomic assignment, financial cost alone would dramatically limit uptake compared to metabarcoding. Additionally, while some bioinformatic skills are required for metabarcoding, the analysis of metagenomic data presents a more complex challenge, and would additionally act as a barrier to uptake. Nevertheless, metagenomics does enable a broader sampling along the genomic axis and could be deployed within a SuperGO (Box 1). Although metabarcoding will remain the dominant approach for quite a while, we have already witnessed transformative sequencing capacity change with existing HTS technology. In this sense, we also view the maintenance of bulk DNA extractions for sites (preferably using nondestructive protocols, e.g., Marquina et al., 2019; Nielsen et al., 2019), or even unprocessed sample replicates, as an important strategy (Jarman et al., 2018). Doing so will ensure that site data can be updated as new HTS developments emerge, promoting long‐term network efficiency and effectiveness.

Discussion within the symposium converged on the opinion that an effective, spatially led terrestrial GO network should place importance on maximizing the global distribution of sampling sites and that these should be strategically united under the umbrella of the single‐locus metabarcoding approach (Figure 1a). Within this framework, we agreed that optimal comparison and integration among independently generated community data sets would be best served by the establishment of harmonized (cf. standardized, see Walters & Scholes, 2017) molecular approaches for site‐based biodiversity surveys. The remainder of the symposium was thus focused on identifying how to collectively build an integrative framework for data generation, and the outcomes of this discussion are summarized in the following section.

## 4 A FRAMEWORK FOR HARMONIZED DATA GENERATION WITHIN A SPATIALLY LED GO NETWORK

Generating biodiversity observations compliant with global data standards, and the need for efforts to develop the associated cyberinfrastructure, were an explicit element of early discussions regarding GOs and a GO network (Davies et al., 2014). Toward this goal, the Biocode Commons (http://biocodecommons.org/) was developed as an open community effort, to provide an open source platform to facilitate GO network conformity to existing biodiversity information standards in the taxonomic (Biodiversity Information Standards Organization, Wieczorek et al., 2012), genomic (Genomic Standards Consortium, Field et al., 2011) and data sharing (ISA Commons and BioSharing community, Field et al., 2009; Sansone et al., 2012) domains. Biocode Commons has helped focus efforts to solve the “metadata gap” associated with public molecular repositories, which has been addressed by the GEOME repository (Deck et al., 2017; Riginos et al., 2020). It allows data contributors to create customized yet standard‐compliant spreadsheets that capture the temporal and geospatial context of each biosample (from incidental point records or inventories, and from Sanger sequences to HTS). GEOME represents a fundamental step toward global integration of DNA sequence‐connected biodiversity data, where scientific reproducibility, synthesis and the potential for DNA sequence reuse are maximized (Riginos et al., 2020).

A spatially led, noncentralized GO network would require bottom‐up growth, and many existing international, national and regional projects or initiatives demonstrate the potential for this through broadly shared goals of generating site‐based biodiversity data using metabarcoding (Table 1). However, in addition to standardization for reporting taxonomic, genomic and metadata, we also see the need for both procedures and best practices for the generation of site‐based metabarcoding data to be similarly standardized. Standardization is fundamental to maximize the potential for integration and global syntheses, as limited integration of genomic approaches in biodiversity observation networks is mainly attributed to the lack of global, standardized and well‐contextualized data sets and accompanying best practices (Canonico et al., 2019; Kahlert et al., 2019).

**TABLE 1:** International, national and regional projects or initiatives with broadly shared goals with the proposed spatially led Genomic Observatories (GO) network and generating site‐based biodiversity (terrestrial nonmicrobial) data using the metabarcoding approach. These initiatives were identified in the symposium with high relevance for building up the framework for harmonized data generation within the GO network

Project/initiative	Website	Institution/entity main holder	Spatial scope	Genomic approach	Target biodiversity fraction	Funding source
BIOSCAN	https://ibol.org/programs/bioscan/	University of Guelph	Global	Community metabarcoding—specimens microbiome metabarcoding	Terrestrial arthropods collected by Malaise traps	University of Guelph
SITE100	https://www.site100.org/	NHM London	Global	Community metabarcoding—mito metagenomics	Terrestrial arthropods collected by interception, pitfall and Malaise traps, soil arthropods extracted by FBF protocol	NHM Biodiversity Initiative
IceCommunities	https://cordis.europa.eu/project/id/772284	Universita degli studi di Milano	Global	eDNA metabarcoding	eDNA (bacteria, fungi, protists, plants, animals) from soil samples	European Research Council
GlobNet	https://leca.osug.fr/ANR‐2021‐2017‐GlobNets	LECA	Global	eDNA metabarcoding	eDNA (bacteria, fungi, protists, plants, animals) from soil samples	ANR (2017–2021)
VIGILIFE	https://beauvalnature.org/en/conservation/programme/spygen‐vigilife	SPYGEN	Global	eDNA metabarcoding	eDNA filtered water samples	WWF, CNRS, Bonneval Nature, Nat Geo, and others
GSSP	https://www.helsinki.fi/en/researchgroups/spatial‐food‐web‐ecology/research/gssp/about‐gssp	University of Helsinki	Global	eDNA metabarcoding	eDNA (fungi) filtered air samples	Academy of Finland
LIFEPLAN	https://www.helsinki.fi/en/projects/lifeplan	University of Helsinki	Global	Community metabarcoding—eDNA metabarcoding	eDNA (bacteria, fungi, protists, plants, animals) from soil samples, eDNA (fungi) filtered air samples, terrestrial arthropods collected by Malaise traps	European Research Council
LUCAS	https://esdac.jrc.ec.europa.eu/projects/lucas	EC Joint Research Centre	Europe	eDNA metabarcoding	eDNA (bacteria, fungi, protists, plants, animals) from soil samples	EC Joint Research Centre
DNAqua‐Net	http://dnaqua.net/	University of Duisburg‐Essen	Europe	Community metabarcoding—eDNA metabarcoding	Aquatic arthropods collected by mesh, eDNA (bacteria, fungi, protists, plants, animals) filtered water samples	COST
1000rivers	https://1000rivers.net/	NatureMetrics	Europe	eDNA metabarcoding—specimens microbiome metabarcoding	eDNA (fish) filtered water samples	NatureMetrics, Citizen groups
Pacific Scanner		Okinawa Institute of Science and Technology	Pacific archipelagos	Community metabarcoding, gut content metabarcoding of individual specimens	Terrestrial arthropods collected by vegetation beating and leaf litter sifting	Okinawa Institute of Science & Technology (OIST) Kick‐start fund
Insect Biome Atlas	https://insectbiomeatlas.org	Swedish Museum of Natural History	Sweden, Madagascar	Community metabarcoding	Terrestrial arthropods collected by Malaise traps and leaf litter sifting, eDNA (arthropods) from soil samples, insect‐associated microbiomes	Knut & Alice Wallenberg Foundation
SCANDNAnet	https://www.syke.fi/en‐US/Research__Development/Research_and_development_projects/Projects/SCANDANnet/SCANDNAnet(47361)	Finnish Environment Institute SYKE	Europe	Community metabarcoding—eDNA metabarcoding	Aquatic arthropods collected by mesh, eDNA (bacteria, fungi, protists, plants, animals) filtered water samples	Nordic Council of Ministers
CALeDNA	https://ucedna.com/	University of California	California	eDNA metabarcoding	eDNA (bacteria, fungi, protists, plants, animals) from soil samples	Catalyst Grant Program
SOILmesoDiv		IPNA‐CSIC, NHM London	Spain, France, Canary Islands, Madeira, Madagascar	Community metabarcoding	Soil arthropods extracted by FBF protocol	The Royal Society, MINECO
BioWide	https://www.gbif.org/es/dataset/3b8c5ed8‐b6c2‐4264‐ac52‐a9d772d69e9f#description	Århus University	Denmark	eDNA metabarcoding	eDNA (bacteria, fungi, protists, plants, animals) from soil samples	Villum Foundation
Dimensions		University of California	Hawaii	Community metabarcoding	Terrestrial arthropods collected by active searching	

Within the marine realm, the recently constituted Global Omics Observatory Network (GLOMICON; https://glomicon.org/) promotes the alignment of protocols and information standards. These derive from the vast accumulated knowledge of large‐scale surveys of ocean waters, to generate a framework of harmonized methods for long‐term marine biodiversity observation (see Canonico et al., 2019). The terrestrial nonmicrobial realm has yet to experience such a development, but the time is right for the establishment of a standardized framework with metabarcoding at its core. The overarching goal of this framework should be to maximize user uptake and the global scale of the network through “harmonization” rather than “standardization” (Walters & Scholes, 2017). For that, various combinations of standards are favoured, and the minimum set of attributes of the framework is constrained, to allow intercalibrations, conversions and sorting of data among GOs. Such a framework could both maximize cross‐GO integration of biodiversity data, and catalyse network growth. A key mechanism for site inclusion within the network should be the adoption of GO data generation standards.

A harmonized framework for metabarcoding data generation within a GO network can build on recent efforts on the harmonization of conventional inventory data. The Humboldt Core (Guralnick et al., 2018) has recently been proposed as a conceptual framework for capturing, in a standardized and general way, core information about processes underpinning inventory work. The objective of the Humboldt Core is to expand biodiversity data set discovery, interoperability and modelling utility for site‐based biodiversity data, a data type essential for the assessment of biodiversity variation in space and time (Guralnick et al., 2018; Jetz et al., 2019). Although mostly focused on conventional inventories, it provides a useful scaffold for the development of a framework for a terrestrial site‐led GO network by extending the philosophy of the Humboldt Core to metabarcoding data. The Humboldt Core places importance on the reporting of the broader scope of site‐based data (spatial, temporal, taxonomic and environmental), and this is to some extent addressed within existing initiatives (GEOME, Deck et al., 2017; Riginos et al., 2020). However, it also emphasizes the need for reporting and harmonizing within the inventory process, methodology and effort. It is here, at the stage of generating biodiversity data, that the major gap for the implementation of the GO network exists, and thus where effort should be focused.

Metabarcoding analyses of site‐based biodiversity are rapidly accumulating, but concern has been raised that the manner of this increase risks losing sight of the challenges in producing high‐quality and reproducible data (Zinger et al., 2019). Indeed, development of best practices and standardized methods for the generation of metabarcoding data has been a recurrent topic in recent years across different communities (Andújar et al., 2018; Orgiazzi et al., 2015; Ransome et al., 2017). Efforts toward harmonization and best practices have been described for some biodiversity fractions, such as microbiomes (Pollock et al., 2018), fungi (Kõljalg et al., 2013) or eDNA (Creer et al., 2016; Taberlet et al., 2018), but are much less developed for many others (e.g., for Metazoa community metabarcoding). The success of microbial initiatives has pivoted on harmonized protocols for microbial/seawater sampling, DNA extraction, library generation or sequencing (e.g., Caporaso et al., 2012; Marotz et al., 2017 for the EMP or Alberti, 2017; Gorsky et al., 2019; Kopf, 2015 for the TARA Oceans and the OSD). Discussion within the symposium identified the following five steps as important for harmonization across the inventory process, methodology and effort. (i) Sample acquisition—including sampling design (spatial scale, temporal scale and biological replicates), sampling method, sampling effort and sample storage. (ii) Sample processing (pre‐DNA extraction)—potentially including protocols for sample cleaning, size selection, photo recording, extraction of vouchers, homogenization and sample storage. (iii) DNA xtraction—DNA extraction protocols, negative and positive controls, technical replicates and long‐term storage. (iv) Amplification, library preparation and sequencing effort—including the target metabarcode fragment(s), sample screening, PCR and library preparation protocols, negative controls, mock standards for intercalibration, sequencing technology and minimum sequencing effort. (v) DNA sequence and metadata sharing and storage (Figure 1b). A challenge for the development of such a framework is that, while existing implementations are in general achievable with a single, or several, type(s) of site‐based community sample (e.g., microscopic communities collected by filtering water/air or eDNA), capturing nonmicrobial biodiversity requires a more diverse sampling and sample processing approach.

We discussed the potentially complex sampling requirements that would typically be needed to capture all nonmicrobial fractions of terrestrial diversity, and concluded that a “modular” structure for data acquisition is the most promising approach. Modules, as basic building blocks of the framework, can provide simple, integrated and interoperable procedures for site‐based characterization of broad biodiversity fractions. An appropriate module design would comprise a minimum set of protocols (or submodules) across the sequence of the five steps described above, which would facilitate the generation of inventory data across a wide taxonomic spectrum (if possible, complete biotas), in a simultaneous, integrated and cost‐efficient way. Some modules would require a combination of multiple independent protocols (submodules) for the sample acquisition step, but a much simpler pipeline for further steps, for example in the case of terrestrial arthropods. However, other modules may be more efficiently executed with a single integrated sample acquisition protocol, but a more diverse structure for sample processing and DNA extraction, for example in the case of soil or freshwater biodiversity inventory (Figure 1b). Common submodules across different modules are also desirable to minimize overall complexity. A modular structure has been successfully incorporated in ForestGEO (https://forestgeo.si.edu), where individual trees are inventoried for multiple layers of biodiversity (e.g., arthropods, vertebrates or lianas) using harmonized pipelines. We propose that similar success can be achieved across broader geographical and environmental scales, using metabarcoding as a shared element across modules designed for specific fractions of terrestrial biodiversity.

Biodiversity fractions for which (i) there is high potential for global integration from site‐based data, and (ii) metabarcoding protocols, data sets or sampling programmes already exist (or are in an advanced stage of development), are obvious candidates for module development. Soil biodiversity emerged as a potential candidate, given that soils host the vast majority of life on Earth, provide ecosystem services that support above‐ground food webs, and are also one of the last “biotic frontiers” of biodiversity (Bardgett & van der Putten, 2014). HTS approaches, and in particular metabarcoding, have been extensively applied to reveal community structure among microbial prokaryotes (bacteria and archaea) and fungi across most soil biomes, including global‐scale initiatives (Delgado‐Baquerizo et al., 2018; Tedersoo, 2014; Thompson et al., 2017). Thus, there is a solid foundation of methodological harmonization and data integration for the development of a soil biodiversity module. The application of metabarcoding to characterize the diversity of soil protist or fauna fractions has been limited (Arribas et al., 2016; Geisen, 2016), and approaches integrating whole soil biota are even more scarce (but see Zinger et al., 2019 using eDNA). Recent efforts to compile a guide of methods for obtaining taxonomic and functional profiles of soil biodiversity have identified metabarcoding as a fundamental tool (Geisen et al., 2019). Microbial, mesofauna, macrofauna and eDNA could be simultaneously sampled within a unified sampling design, including a combination of sample processing protocols to extract bulk communities (e.g., arthropods, nematodes, roots) and eDNA from the soil matrix. Methodological and logistical advances within ongoing large‐scale soil biodiversity projects such as the LUCAS, GlobNet, LIFEPLAN or IceCommunities (see Table 1 for details) could be used to develop a soil biodiversity module.

Terrestrial arthropods also emerged as a biodiversity fraction with high potential for a modular approach, being a hyperdiverse and ubiquitous group with high potential for the application of HTS (Ji et al., 2013; Yu et al., 2012). Best practices and harmonization for metabarcoding of bulk arthropod samples are still in the early stages of development, but already there are multiple global initiatives that pivot on arthropod metabarcoding (e.g., the BIOSCAN initiative and its regional extensions such as the BioAlfa or the Kruger Malaise Program, the SITE‐100 project, the Insect Biome Atlas Project, and LIFEPLAN; see Table 1). We view this critical mass, which is complemented by various national‐level efforts, as a solid platform for the development of a terrestrial arthropod module, where passive sampling methods (e.g., pitfall, light and malaise traps, the last of which is already standardized within the BIOSCAN initiative) can be implemented individually, or in combination. Recent efforts toward developing best practices for COI community metabarcoding (e.g., Andújar et al., 2018; Elbrecht et al., 2019; Marquina et al., 2019) can be further developed for harmonized protocols for subsequent sample processing.

## 5 CONCLUDING REMARKS

The value of large‐scale site‐based records for terrestrial invertebrates has been recently demonstrated for nematodes (van den Hoogen et al., 2019) and earthworms (Phillips et al., 2019), where synthetic analyses have yielded predictions of global diversity patterns within both groups. However, for other fractions of biodiversity, new initiatives are needed to provide such data, which presents both a challenge and at the same time an opportunity. We conclude that the establishment of an integrative HTS framework for measuring and understanding global patterns of biodiversity is now a realizable ambition. We further conclude that, by placing metabarcoding within the core of this framework, there is high potential for: (i) the establishment of harmonized protocols; (ii) fostering participation; (iii) capitalizing upon recent initiatives; and (iv) comparability and integration of data among network members. While work continues to address remaining challenges for some biodiversity metrics, such as abundance and biomass (Bista et al., 2018; Lamb et al., 2019), these challenges are balanced by unique opportunities from DNA‐based biodiversity data. These include automated taxonomic assignment, spatial maps of beta diversity, and the ability to integrate phylogeny and genetic diversity with other environmental variables to understand not only how biodiversity is structured globally, but also the overarching processes that might explain the dynamics of community assembly (e.g., Theodoridis et al., 2020). Within such a framework, deeper genomic and temporal sampling can be developed within sites of interest, building upon the foundation of local metabarcode data, and the spatial context of broader regional and global sampling within the network.

## AUTHOR CONTRIBUTIONS

All authors contributed to the ideas and discussion of this review. P.A. and B.C.E. coordinated the Symposium and led the writing with contributions from all authors.

## Data Availability

No data were analysed in this meeting review.
